# Status of COVID-19 Patients Treated With Extracorporeal Membrane Oxygenation in Japan: Nationwide Database Analysis

**DOI:** 10.7759/cureus.60202

**Published:** 2024-05-13

**Authors:** Tomoki Kuribara, Yusuke Asai, Norio Ohmagari, Isao Yokota

**Affiliations:** 1 Biostatistics, Graduate School of Medicine, Hokkaido University, Sapporo, JPN; 2 Acute and Critical Care Nursing, School of Nursing, Sapporo City University, Sapporo, JPN; 3 Antimicrobial Resistance (AMR) Clinical Reference Center, National Center for Global Health and Medicine, Tokyo, JPN; 4 Disease Control and Prevention Center, National Center for Global Health and Medicine, Tokyo, JPN

**Keywords:** icu (intensive care unit), pulmonary critical care, extensive registry database, covid-19, ecmo

## Abstract

Background

The report of epidemiological data on coronavirus disease 2019 (COVID-19) patients treated using extracorporeal membrane oxygenation (ECMO) in Japan has been limited. Our study seeks to fill the existing gap in knowledge by providing an in-depth analysis of the clinical epidemiological characteristics and diverse medical outcomes of COVID-19 patients treated with ECMO in Japan.

Methods

This study used the COVID-19 Registry Japan nationwide database. We included patients aged 18 years or older enrolled between March 17, 2020, and February 1, 2022, with traceable ECMO data. The items on clinical epidemiological characteristics and various medical outcomes were collected. Statistical analysis included a median and interquartile range (IQR) for continuous variables and frequencies for categorical variables.

Results

The number of participating hospitals was 731, and the number of patients enrolled for analysis was 49,590. Of these, 196 (0.4%) patients received ECMO. Hospital mortality was 33.2%, and discharge to home was 23.0% in the ECMO group. The complications during hospitalization included pneumothorax (9.7%), seizures (4.1%), stroke (4.6%), and pulmonary thromboembolism (2.0%). At discharge, 38.3% had worsened self-care ability, and 38.8% had worsened ambulatory function.

Conclusions

The results of ECMO treatment in Japan showed that the mortality and complication rates were well-controlled compared with those worldwide.

## Introduction

Since 2019, the outbreak of coronavirus disease 2019 (COVID-19) has escalated into a global pandemic, presenting unprecedented challenges to healthcare systems worldwide. Among the myriad of complications associated with COVID-19, severe respiratory failure stands out, often requiring intensive care [1], like extracorporeal membrane oxygenation (ECMO), which has been a critical tool in managing severe respiratory failure worldwide, with its efficacy well-established [2].

Extracorporeal membrane oxygenation has also been used for severe respiratory failure in COVID-19 cases worldwide. However, a notable variability in outcomes has been observed, influenced by regional and institutional differences [3-4]. This variability can be attributed to factors such as the rapid and extensive spread of the virus, the evolving efficacy of treatments for severe respiratory failure [5], and the diverse results observed by different countries. Various circumstances emerged worldwide, with some nations successfully implementing measures while others faced challenges. Furthermore, the COVID-19 virus underwent mutations with varying characteristics in various locales [6], highlighting the necessity for region-specific medical systems.

In Japan, the pandemic has led to a significant reliance on ECMOs for managing severe respiratory failure [7]. However, the reported outcomes for COVID-19 patients nationwide in Japan have been limited to survival rates [8], a metric that, while important, does not fully encapsulate the multifaceted nature of effective medical treatment. In contrast, other countries have provided more comprehensive reports, encompassing a range of clinical and epidemiological outcomes [3,9]. Such detailed reporting is invaluable, contributing significantly to the global understanding of respiratory infectious diseases and their management.

In light of this, our study seeks to fill the existing gap in knowledge by providing an in-depth analysis of the clinical epidemiological characteristics and diverse medical outcomes of COVID-19 patients treated with ECMO in Japan. This approach not only aligns with the global need for comprehensive data on COVID-19 management but also contributes uniquely to the body of knowledge on the effectiveness of ECMO in varied clinical settings.

## Materials and methods

Study design and data source

This study was a retrospective cross-sectional study using the COVID-19 Registry Japan (COVIREGI-JP), an extensive registry database of patients at various sites during the COVID-19 pandemic. The study data were collected and managed using REDCap (Research Electronic Data Capture), a secure, web-based data capture application hosted at the Joint Center for Researchers, Associates, and Clinicians (JCRAC) data center of the National Center for Global Health and Medicine. All patients registered in COVIREGI-JP were diagnosed with COVID-19. Extensive data were collected on their life background before admission, vaccination status for COVID-19, drugs used during hospitalization, and treatment during hospitalization. This study was conducted with the approval of the ethics committee of Hokkaido University Hospital, Sapporo, Japan (approval number: 021-0211) and COVIREGI-JP (approval number: 1248600101). The study conducted has been reported in accordance with the Strengthening the Reporting of Observational Studies in Epidemiology (STROBE) statement [10].

Patient selection

Data from patients over 18 years enrolled between March 17, 2020, when COVIREGI-JP was opened, and February 1, 2022, were included. Patients with missing data on ECMO implementation during hospitalization were excluded from the analysis. Only those subjects for whom responses regarding ECMO implementation during hospitalization were available were classified into the ECMO group. For ECMO implementation responses, COVIREGI-JP does not distinguish between venovenous (V-V) ECMO and venoarterial (V-A) ECMO. Therefore, all patients who used ECMO, whether V-V or V-A, were included in response to ECMO implementation.

Collected items

As for the clinical epidemiological characteristics, patient background information like their age, sex, smoking history, drinking alcohol, and body mass index (BMI) was collected. In addition, as conditions at admission, the following items were collected: days from symptom onset to hospitalization, body temperature, heart rate, respiratory rate, systolic blood pressure, diastolic blood pressure, state of consciousness (alert, verbal, pain, and unresponsive (AVPU) scale), oxygen saturation (SpO_2_) under room air, route of oxygen administration (canula, mask, reservoir mask, high flow oxygen device), chest X-ray at admission (performed within ±3 days of admission), CT at admission (performed within ±3 days of admission). Furthermore, significant comorbidities at the time of hospitalization and other items related to major treatments performed during hospitalization were collected.

The medical outcomes in this study were defined as the patient outcomes at hospital discharge, the incidence of complications during hospitalization (pneumothorax, seizure, intracerebral bleeding/ischemic stroke, pulmonary thromboembolism), the patient's condition during hospital discharge regarding self-care ability, and ambulatory function. The complications of stroke included both cerebral hemorrhage and cerebral infarction.

Statistical analysis

Continuous variables were described as the median and interquartile range (IQR), and categorical variables were calculated for their frequencies. The number of deaths and the count of patients who worsened or required assistance with self-care ability and those who worsened or required assistance with ambulatory function in the ECMO group are shown against age. The percentage of missing values was calculated for each item when the items had missing values. All statistical analyses were conducted using R version 4.1.2 (R Foundation for Statistical Computing, Vienna, Austria).

## Results

During the study period, 51,441 patients were enrolled, and 731 hospitals participated. Of these, 49,590 patients were included in the analysis as participants, after excluding those with missing responses regarding ECMO implementation during hospitalization. Among the participants, 196 patients (0.4%) were in the ECMO group.

Characteristics of patients

Participant characteristics are listed in Table 1. The median age of all patients was 58 years (IQR, 42-74 years), with the ECMO group being 59.5 years (51-58 years) and the non-ECMO group being 58 years (IQR, 42-74 years). Of all the patients, 28,664 (57.7%) were male, with 160 patients (81.6%) in the ECMO group and 28,504 patients (57.7%) in the non-ECMO group being male. The median BMI of all patients was 23.6 kg/m^2^ (IQR, 21-26.7 kg/m^2^), with the ECMO group having a median BMI of 27.7 kg/m^2^ (IQR, 24.5-31.1 kg/m^2^) and the non-ECMO group having a median BMI of 23.6 kg/m^2^ (IQR, 21-26.7 kg/m2).

**Table 1:** Characteristics of the patients Values are presented as median (interquartile range) or n(%). BMI: body mass index; SpO2: oxygen saturation; CT: computed tomography; COPD: chronic obstructive pulmonary disease; ECMO: extracorporeal membrane oxygenation. ^a^Percentage showed less than 0.1%. ^b^Immunosuppression includes neutropenia (<500 neutrophils/μL), glucocorticoid/steroid use within one month (doses greater or equal to an equivalent of 20 mg of prednisone per day for at least one month), chemotherapy, radiation therapy, or immunosuppressant use (such as anti-tumor necrosis factor α therapy, anti-interleukin-6 receptor/anti-CD20 monoclonal antibodies, selective T-cell co-simulation blocker, methotrexate, tacrolimus) in the past three months, post-transplantation, asplenia, and primary immunodeficiency syndrome).

Parameters	Subcategories	Overall (n=49590)	ECMO (n=196)	Non-ECMO (n=49394)
Background				
Age (years)		58.0 (42.0, 74.0)	59.5 (51.0, 68.0)	58.0 (42.0, 74.0)
Sex	Male	28664 (57.8)	160 (81.6)	28504 (57.7)
	Female	20909 (42.2)	36 (18.4)	20873 (42.3)
	Other	13 (0.0) ^a^	0	13 (0.0) ^a^
Smoking history	Currently smoking	7936 (16.0)	26 (13.3)	7910 (16.0)
	Smoking in the past	10689 (21.6)	66 (33.7)	10623 (21.5)
	Never	22799 (46.0)	65 (33.2)	22734 (46.0)
	Unknown	8068 (16.3)	39 (19.9)	8029 (16.3)
Drinking alcohol	Daily	3146 (6.3)	11 (5.6)	3135 (6.3)
	Occasional	16247 (32.8)	63 (32.1)	16184 (32.8)
	None	17496 (35.3)	45 (23.0)	17451 (35.3)
	Unknown	12059 (24.3)	70 (35.7)	11989 (24.3)
BMI (kg/m^2^)		24.0 (21.0, 26.7)	27.7 (24.5, 31.1)	23.6 (21.0, 26.7)
Conditions at admission				
Days from symptom onset to hospitalization		4.0 (2.0, 7.0)	7.0 (4.0, 10.0)	4.0 (2.0, 7.0)
Body temperature (℃)		37.0 (36.6, 37.8)	37.5 (36.8, 38.3)	37.0 (36.6, 37.8)
Heart rate (beats/minute)		86.0 (76.0, 97.0)	94.0 (79.8, 107.3)	86.0 (76.0, 97.0)
Respiratory rate (breaths/minute)		18.0 (16.0, 21.0)	22.0 (18.0, 28.0)	18.0 (16.0, 21.0)
Systolic blood pressure (mmHg)		128.0 (115.0, 142.0)	130.0 (112.0, 147.8)	128.0 (115.0, 142.0)
Diastolic blood pressure (mmHg)		79.0 (70.0, 89.0)	78.0 (68.0, 90.0)	79.0 (70.0, 89.0)
State of consciousness (AVPU scale)	A (alert)	45742 (92.2)	138 (70.4)	45604 (92.3)
	V (verbal)	1376 (2.8)	17 (8.7)	1359 (2.8)
	P (pain)	261 (0.5)	6 (3.1)	255 (0.5)
	U (unresponsive)	145 (0.3)	8 (4.1)	137 (0.3)
SpO_2_ under room air (%)		97.0 (95.0, 98.0)	94.0 (90.0, 96.0)	97.0 (95.0, 98.0)
Route of noninvasive O_2_ administration	Nasal cannula	5429 (10.9)	17 (8.7)	5412 (11.0)
	Face mask	1526 (3.1)	30 (15.3)	1496 (3.0)
	Reservoir mask	1074 (2.2)	28 (14.3)	1046 (2.1)
	High-flow oxygen device	210 (0.4)	6 (3.1)	204 (0.4)
Finding by X-ray	No abnormality	14494 (29.2)	8 (4.1)	14486 (29.3)
	Pneumonia	20008 (40.3)	164 (83.7)	19844 (40.2)
	Abnormality (excluding pneumonia)	558 (1.1)	1 (0.5)	557 (1.1)
Finding by CT	No abnormality	7591 (15.3)	4 (2.0)	7587 (15.4)
	Pneumonia	28031 (56.5)	157 (80.1)	27874 (56.4)
	Abnormality (excluding pneumonia)	886 (1.8)	4 (2.0)	882 (1.8)
Comorbidities				
Myocardial infarction		946 (1.9)	6 (3.1)	940 (1.9)
Congestive heart failure		1567 (3.2)	4 (2.0)	1563 (3.2)
Cerebrovascular disease		3231 (6.5)	9 (4.6)	3222 (6.5)
Paralysis		725 (1.5)	0	725 (1.5)
COPD		1214 (2.4)	9 (4.6)	1205 (2.4)
Chronic lung disease other than COPD		711 (1.4)	6 (3.1)	705 (1.4)
Bronchial asthma		2652 (5.3)	15 (7.7)	2637 (5.3)
Moderate to severe liver dysfunction		169 (0.3)	1 (0.5)	168 (0.3)
Hypertension		15336 (30.9)	82 (41.8)	15254 (30.9)
Dyslipidemia		7312 (14.7)	43 (21.9)	7269 (14.7)
Diabetes with complications		1108 (2.2)	8 (4.1)	1100 (2.2)
Obesity		3501 (7.1)	37 (18.9)	3464 (7.0)
Moderate to severe renal dysfunction		833 (1.7)	4 (2.0)	829 (1.7)
Hemodialysis before admission		495 (1.0)	2 (1.0)	493 (1.0)
Immunosuppression^b^		1050 (2.1)	4 (2.0)	1046 (2.1)

Treatments performed during hospitalization

During hospitalization, 599 (1.2%) patients received noninvasive mechanical ventilation. Among them, 32 (16.3%) belonged to the ECMO group, while 567 (1.1%) belonged to the non-ECMO group. In terms of invasive mechanical ventilation, 2,439 (4.9%) overall patients received it, with 192 (98.0%) in the ECMO group and 2,247 (4.5%) in the non-ECMO group. The median length of ICU stay was seven days (IQR, 3-14 days) for overall patients, 21 days (IQR, 14-35 days) for the ECMO group, and seven days (IQR, 3-13 days) for the non-ECMO group. The median duration of ECMO treatment was 11 days (IQR, 8-20 days). Table 2 provides further details.

**Table 2:** Patient status during hospitalization Values are presented as median (interquartile range) or n(%). ECMO: extracorporeal membrane oxygenation; ARDS: acute respiratory distress syndrome; RRT: renal replacement therapy; NA: not available ^a^Noninvasive mechanical ventilation includes biphasic positive airway pressure (BIPAP) or continuous positive airway pressure). ^b^Data included only the patients who were alive at discharge.

Parameters	Subcategories	Overall (n=49590)	ECMO (n=196)	Non-ECMO (n=49394)
Treatments during hospitalization				
Noninvasive mechanical ventilation^a^		599 (1.2)	32 (16.3)	567 (1.1)
Mechanical ventilation		2439 (4.9)	192 (98.0)	2247 (4.5)
Mechanical ventilation duration days		8.0 (4.0, 14.0)	16.0 (11.0, 28.0)	7.0 (4.0, 13.0)
Days to ECMO initiation from admission		4.0 (1.0, 8.0)	4.0 (1.0, 8.0)	NA
Length of ICU stay (days)		7.0 (3.0, 14.0)	21.0 (14.0, 35.0)	7.0 (3.0, 13.0)
ECMO duration days		11.0 (8.0, 20.0)	11.00 (8.0, 20.0)	NA
Prone positioning		2302 (4.6)	105 (53.6)	2197 (4.4)
Nitric oxide inhalation		37 (0.1)	9 (4.6)	28 (0.1)
Tracheostomy		426 (0.9)	69 (35.2)	357 (0.7)
Neuromuscular blocking agent		1415 (2.9)	140 (71.4)	1275 (2.6)
Vasopressor support		1339 (2.7)	141 (71.9)	1198 (2.4)
RRT or dialysis		706 (1.4)	66 (33.7)	640 (1.3)
Blood transfusion		979 (2.0)	158 (80.6)	821 (1.7)
Complications				
ARDS		2323 (4.7)	117 (59.7)	2206 (4.5)
Severity of ARDS	Mild	499 (1.0)	3 (1.5)	496 (1.0)
	Moderate	789 (1.6)	21 (10.7)	768 (1.6)
	Severe	969 (2.0)	89 (45.4)	880 (1.8)
Myocardial ischemia		87 (0.2)	5 (2.6)	82 (0.2)
Bacteremia		474 (1.0)	53 (27.0)	421 (0.9)
Gastrointestinal bleeding		290 (0.6)	20 (10.2)	270 (0.5)
Patient status at discharge				
Oxygen therapy required^b^		3231 (6.5)	34 (17.3)	3197 (6.5)
RRT or dialysis^b^		377 (0.8)	3 (1.5)	374 (0.8)
Tracheostomy^b^		362 (0.7)	18 (9.2)	344 (0.7)

Medical outcomes

**Figure 1:**
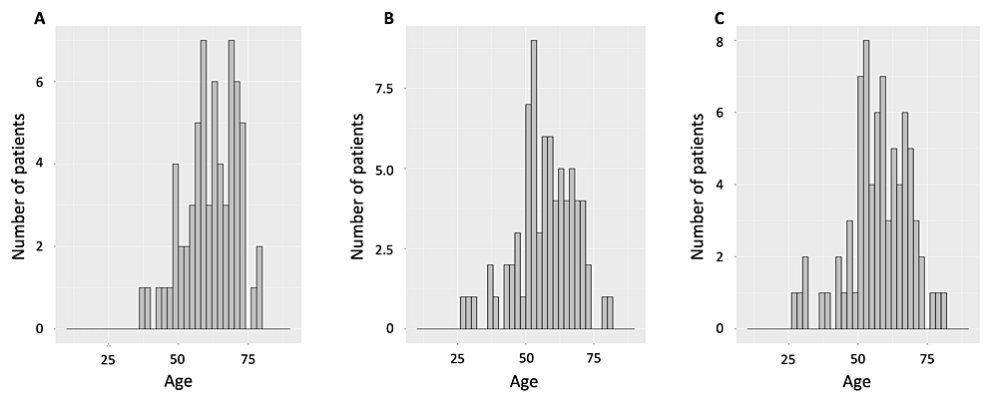
The distribution of patients by outcomes in the ECMO group A: The distribution of hospital mortality in the ECMO group indicates that the number of patients who died increased in the late 1950s. B: The distribution of worsened or required assistance with self-care ability in the ECMO group showed patients increased from the early 50s. C: The distribution of worsened or required assistance with ambulatory function in the ECMO group showing patients increased from the early 50s. ECMO: extracorporeal membrane oxygenation

The results of the medical outcomes are shown in Table 3. This study found that hospital mortality was higher in the ECMO group (33.2%) compared to the non-ECMO group (4.8%). Regarding other patient outcomes, more patients in the ECMO group (23.0%) were discharged to acute care hospitals compared to the non-ECMO group (11.6%). A lower percentage of patients in the ECMO group (0.5%) were discharged to long-term care centers than the non-ECMO group (4.0%). The majority of patients (73.8%) were discharged home. The complications during hospitalization were pneumothorax (9.7%), seizures (4.1%), stroke (4.6%), and pulmonary thromboembolism (2.0%) in the ECMO group. In addition, at the time of discharge, a higher percentage of patients in the ECMO group (38.3%) had worsened or required assistance with self-care ability compared to the non-ECMO group (8.7%). Similarly, a higher percentage of patients in the ECMO group (38.8%) had worsened or required assistance with ambulatory function compared to the non-ECMO group (9.0%). This study also provided information on the age distribution of patients in the ECMO group who died, which increased beginning in the late 50s, and patients with worsened or required assistance with self-care ability and ambulatory function increased from the early 50s (Figure 1). The number of missing data points for all variables has been provided in Appendices A-C.

**Table 3:** Medical outcomes Values are presented as n(%). ECMO: extracorporeal membrane oxygenation ^a^Still in the hospital after 60 days from admission. ^b^Percentage showed less than 0.1%. ^c^Data included only the patients who were alive at discharge.

Parameters	Subcategories	Overall (n=49590)	ECMO (n=196)	Non-ECMO (n=49394)
Patient outcomes at hospital discharge				
Hospital mortality, %(n)		4.9 (2437)	33.2 (65)	4.8 (2372)
Discharge to home		36598 (73.8)	45 (23.0)	36553 (74.0)
Discharge to long-term care center		1962 (4.0)	1 (0.5)	1961 (4.0)
Discharge to acute care hospital		5798 (11.7)	57 (29.1)	5741 (11.6)
Discharge to an isolation hospital		2132 (4.3)	0	2132 (4.3)
Still in the hospital^a^		11 (0.0)^b^	1 (0.5)	10 (0.0)^b^
Complications				
Pneumothorax		201 (0.4)	19 (9.7)	182 (0.4)
Seizures		111 (0.2)	8 (4.1)	103 (0.2)
Stroke		175 (0.4)	9 (4.6)	166 (0.3)
Pulmonary thromboembolism		170 (0.3)	4 (2.0)	166 (0.3)
Patient condition at discharge				
Self-care ability^c^	Same as before hospitalization	40098 (80.9)	22 (11.2)	40076 (81.1)
	Worsened or required assistance	4393 (8.9)	75 (38.3)	4318 (8.7)
	Improved	990 (2.0)	1 (0.5)	989 (2.0)
	Unknown	3099 (6.2)	75 (38.2)	3024 (6.1)
Ambulatory function^c^	Same as before hospitalization	39428 (79.5)	18 (9.2)	39410 (79.8)
	Worsened or required assistance	4507 (9.1)	76 (38.8)	4431 (9.0)
	Improved	795 (1.6)	1 (0.5)	794 (1.6)
	Unknown	901 (1.8)	6 (3.1)	895 (1.8)

## Discussion

Our study utilized the COVIREGI-JP database to conduct a descriptive analysis of COVID-19 patients in Japan treated with ECMO. We identified the mortality rate and the range of complications associated with ECMO treatment. Notably, our findings show a mortality rate of about 33% in Japan, consistent with national data [8] and slightly more favorable outcomes compared to international meta-analyses [3-4].

A significant aspect of our study was examining the age demographics of patients treated with ECMO in Japan. We observed that the third quartile of age was higher compared to other countries [9, 11] but younger than the overall group. This suggests a more selective approach to ECMO use in non-elderly patients within Japan, potentially contributing to the slightly better mortality outcomes observed. Our findings align with existing research indicating that age is a significant factor in ECMO outcomes, with increased mortality risks associated with ECMO in patients over the age of 59 [8-9]. This selective approach may reflect a balance between the risks and benefits of ECMO treatment in an aging population, highlighting the importance of considering patient age in ECMO treatment decisions.

The incidence of complications in the ECMO group is managed at the same level worldwide. In general, significant complications related to ECMO treatment are reported to be related to hemorrhage [12], just as in COVID-19 patients [13-14]. For intracerebral hemorrhage, many studies have reported incidence rates ranging from 2.3% to 17.4% [9,11,15-21], with some reporting incidence rates as high as 41.7% [22]. In addition, ischemic stroke has been reported to have an incidence of 0.7％ to 7％ [9,11,15-16,18-21]. These results suggest that the incidence of intracerebral hemorrhage and ischemic stroke for patients managed with ECMO in Japan is comparable to other countries. Regarding the incident rate of seizures, the previous study reported 0% to 2% [9,18,20] and a rise to 14% restricted to non-survivors [18]. Our study included both non-survivors and survivors, which may have resulted in a higher percentage. In the development of pneumothorax, previous studies reported the incidence rate was 11％ and 12.6％ [15,23], and the present results were similar to slightly lower rates. For pulmonary thromboembolism, various studies have reported incidence rates ranging from 1.6% to 19% [11,15-18,23-24], with some reporting rates of over 60% [25-26]. In light of these circumstances, the incidence of pulmonary thromboembolism in Japan is confirmed to be relatively low. A unique ECMO support system was established in Japan to assist almost all ICUs treating COVID-19 patients who require ECMO [27]. Furthermore, the Japan ECMOnet for COVID-19, a collaboration of several major Japanese societies specializing in ECMO, has introduced Japan’s basic ECMO management concept [28]. The results of this study may reflect the variety of Japan’s responses to the COVID-19 pandemic.

Finally, this study indicates the significant impact on post-discharge life, particularly for patients in their post-middle-age years, underscoring the need for greater attention to these effects. Compared to those hospitalized in general wards, there was a decline in activities of daily living (ADL) after hospital discharge in older adult patients with COVID-19 who were admitted to the ICU [29]. Additionally, a reduction in ambulatory function at discharge was linked to an ongoing functional decline post-discharge [30]. The implications of these results may not be limited to Japan, as the rate of home discharge for patients who used ECMO in Japan was similar to that in other countries [9].

However, our study has limitations. The COVIREGI-JP lacks specific ICU data, including detailed invasive mechanical ventilator management and ECMO types like V-V ECMO or V-A ECMO, which restricts our ability to provide a comprehensive analysis of ICU treatments and outcomes.

Research implication

Most registry databases aim to collect data during hospitalization, and medical records do not include post-discharge data. We need the database collecting post-discharge data or other study methodologies to understand the daily lives of patients post discharge. In addition, post-discharge studies on COVID-19 patients who used ECMO are limited, and the cohort is small. Therefore, we may need a prospective study targeting a large cohort or large number to explore the effect and provide more evidence of the post-discharge life of COVID-19 patients on whom ECMO was used.

## Conclusions

In this study, we revealed the clinical epidemiological characteristics of COVID-19 patients treated with ECMO. The results of this study suggest that Japanese hospitals managed COVID-19 patients with a more selective approach to ECMO use in cases of non-elderly patients. The medical outcomes related to ECMO-treated COVID-19 patients, such as hospital mortality and incidence of complications, were as good as or somewhat better than their counterparts in other countries. Furthermore, the results of this study indicate a potential impact on post-discharge life, particularly in post-middle-age patients. Future research should focus on the post-discharge life of ECMO patients to provide more evidence, as a wide range of ages may be affected by post-discharge life.

## Appendices

Appendix A 

**Table 4:** Number of missing data in characteristics of patients Values are presented as n(%). BMI: body mass index; SpO2: oxygen saturation; CT: computed tomography; COPD: chronic obstructive pulmonary disease; ECMO: extracorporeal membrane oxygenation ^a^Percentage showed less than 0.1%. ^b^Immunosuppression includes neutropenia (<500 neutrophils/μL), glucocorticoid/steroid use within one month (doses greater or equal to an equivalent of 20 mg of prednisone per day for at least one month), chemotherapy, radiation therapy, or immunosuppressant use (such as anti-tumor necrosis factor α therapy, anti-interleukin-6 receptor/anti-CD20 monoclonal antibodies, selective T-cell co-simulation blocker, methotrexate, tacrolimus) in the past three months, post-transplantation, asplenia, primary immunodeficiency syndrome.

Parameters	Overall (n=49590)	ECMO (n=196)	Non-ECMO (n=49394)
Background			
Sex	4 (0.0) ^a^	0	4 (0.0) ^a^
Smoking history	98 (0.2)	0	98 (0.2)
Drinking alcohol	642 (1.3)	7 (3.6)	635 (1.3)
BMI (kg/m^2^)	7993 (16.0)	16 (8.0)	7977 (16.0)
Conditions at admission			
Days from symptom onset to hospitalization	3924 (7.9)	8 (4.0)	3916 (7.9)
Body temperature (℃)	81 (0.2)	0	81 (0.2)
Heart rate, median (beats/minute)	1002 (2.0)	0	1002 (2.0)
Respiratory rate (breaths/minute)	12580 (25.4)	22 (11.0)	12558 (25.4)
Systolic blood pressure (mmHg)	1054 (2.1)	2 (1.0)	1052 (2.1)
Diastolic blood pressure (mmHg)	1117 (2.3)	3 (2.0)	1114 (2.3)
State of consciousness (AVPU scale)	2066 (4.2)	27 (13.8)	2039 (4.1)
SpO_2_ under room air (%)	279 (0.6)	0	279 (0.6)
Route of noninvasive O_2_ administration	41351 (83.4)	115 (58.7)	41236 (83.5)
Finding by X-ray	14530 (29.3)	23 (11.7)	14507 (29.4)
Finding by CT	13082 (26.4)	31 (15.8)	13051 (26.4)
Comorbidities			
Myocardial infraction	525 (1.1)	22 (11.2)	503 (1.0)
Congestive heart failure	525 (1.1)	22 (11.2)	503 (1.0)
Cerebrovascular disease	525 (1.1)	22 (11.2)	503 (1.0)
Paralysis	525 (1.1)	22 (11.2)	503 (1.0)
COPD	525 (1.1)	22 (11.2)	503 (1.0)
Chronic lung disease other than COPD	525 (1.1)	22 (11.2)	503 (1.0)
Bronchial asthma	525 (1.1)	22 (11.2)	503 (1.0)
Moderate to severe liver dysfunction	525 (1.1)	22 (11.2)	503 (1.0)
Hypertension	525 (1.1)	22 (11.2)	503 (1.0)
Dyslipidemia	525 (1.1)	22 (11.2)	503 (1.0)
Diabetes with complications	525 (1.1)	22 (11.2)	503 (1.0)
Obesity	525 (1.1)	22 (11.2)	503 (1.0)
Moderate to severe renal dysfunction	525 (1.1)	22 (11.2)	503 (1.0)
Hemodialysis before admission	525 (1.1)	22 (11.2)	503 (1.0)
Immunosuppression^b^	1609 (3.2)	31 (15.8)	1578 (3.2)

Appendix B 

**Table 5:** Number of missing data in patient status during hospitalization Values are presented as n(%). ECMO: extracorporeal membrane oxygenation; ICU: intensive care unit; RRT: renal replacement therapy; ARDS: acute respiratory distress syndrome; NA: not available ^a^Noninvasive mechanical ventilation includes BIPAP (biphasic positive airway pressure) or CPAP (continuous positive airway pressure). ^b^Percentage showed less than 0.1%. ^c^If the patients were not eligible for each of these, they were counted as missing data.

Parameters	Overall (n=49590)	ECMO (n=196)	Non-ECMO (n=49394)
Treatments doing hospitalization			
Noninvasive mechanical ventilation^a^	23 (0.0) ^b^	2 (1.0)	21 (0.0) ^b^
Mechanical ventilation	66 (0.1)	0	66 (0.1)
Mechanical ventilation duration days^c^	47300 (95.0)	9 (5.0)	47291 (96.0)
Days to ECMO initiation from admission^c^	49399 (99.6)	5 (3.0)	NA
Length of ICU stay (days)^c^	45566 (92.0)	2 (1.0)	45564 (92.0)
ECMO duration days^c^	49401 (99.6)	7 (4.0)	NA
Prone positioning	2820 (5.7)	4 (2.0)	2816 (5.7)
Nitric oxide inhalation	2790 (5.6)	6 (3.1)	2784 (5.6)
Tracheostomy	2778 (5.6)	3 (1.5)	2775 (5.6)
Neuromuscular blocking agent	2901 (5.8)	13 (6.6)	2888 (5.8)
Vasopressor support	116 (0.2)	7 (3.6)	109 (0.2)
RRT or dialysis	107 (0.2)	3 (1.5)	104 (0.2)
Blood transfusion	107 (0.2)	1 (0.5)	106 (0.2)
Complications			
Severity of ARDS^c^	47333 (95.4)	83 (42.3)	47250 (95.7)
Myocardial ischemia	931 (1.9)	28 (14.3)	903 (1.8)
Bacteremia	948 (1.9)	30 (15.3)	918 (1.9)
Gastrointestinal bleeding	954 (1.9)	28 (14.3)	926 (1.9)
Patient status at discharge			
Oxygen therapy required	3301 (6.7)	93 (47.4)	3208 (6.5)
RRT or dialysis	3304 (6.7)	93 (47.4)	3211 (6.5)
Tracheostomy	3314 (6.7)	93 (47.4)	3221 (6.5)

Appendix C 

**Table 6:** Number of missing data in medical outcomes Values are presented as n(%). ECMO: extracorporeal membrane oxygenation ^a^Including the value of hospital mortality, discharge to home, discharge to a long-term care center, discharge to acute care hospital, discharge to an isolation hospital, and still in the hospital.

Parameters	Overall (n=49590)	ECMO (n=196)	Non-ECMO (n=49394)
Complications			
Pneumothorax	941 (1.9)	28 (14.3)	913 (1.8)
Seizures	927 (1.9)	28 (14.3)	899 (1.8)
Stroke	919 (1.9)	28 (14.3)	891 (1.8)
Pulmonary thromboembolism	1556 (3.1)	37 (18.9)	1519 (3.1)
Patient condition at discharge			
Self-care ability	3358 (6.8)	93 (47.4)	3265 (6.6)
Ambulatory function	3959 (8.0)	95 (48.5)	3864 (7.8)
Patient outcomes at hospital discharge^a^	652 (1.3)	27 (13.8)	625 (1.3)
